# The effect of two selective A_1_‐receptor agonists and the bitopic ligand VCP746 on heart rate and regional vascular conductance in conscious rats

**DOI:** 10.1111/bph.14870

**Published:** 2020-01-01

**Authors:** Samantha L. Cooper, Julie March, Andrea R. Sabbatini, Stephen J. Hill, Manuela Jörg, Peter J. Scammells, Jeanette Woolard

**Affiliations:** ^1^ Division of Physiology, Pharmacology and Neuroscience, School of Life Sciences University of Nottingham Nottingham UK; ^2^ Centre of Membrane Proteins and Receptors University of Birmingham and University of Nottingham The Midlands UK; ^3^ Medicinal Chemistry, Monash Institute of Pharmaceutical Sciences Monash University Parkville Victoria Australia

## Abstract

**Background and Purpose:**

Adenosine is a local mediator that regulates physiological and pathological processes via activation of four GPCRs (A_1_, A_2A_, A_2B_, and A_3_). We have investigated the effect of two A_1_‐receptor‐selective agonists and the novel A_1_‐receptor bitopic ligand VCP746 on the rat cardiovascular system.

**Experimental Approach:**

The regional haemodynamic responses of these agonist was investigated in conscious rats. Male Sprague–Dawley rats (350–450 g) were chronically implanted with pulsed Doppler flow probes on the renal, mesenteric arteries and the descending abdominal aorta and the jugular vein and caudal artery catheterized. Cardiovascular responses were measured following intravenous infusion (3 min each dose) of CCPA (120, 400, and 1,200 ng·kg^−1^·min^−1^), capadenoson or adenosine (30, 100, and 300 μg·kg^−1^·min^−1^), or VCP746 (6, 20, and 60 μg·kg^−1^·min^−1^) following pre‐dosing with DPCPX (0.1 mg·kg^−1^, i.v.) or vehicle.

**Key Results:**

CCPA produced a significant A_1_‐receptor‐mediated decrease in heart rate that was accompanied by vasoconstrictions in the renal and mesenteric vascular beds but an increase in hindquarters vascular conductance. The partial agonist capadenoson also produced an A_1_‐receptor‐mediated bradycardia. In contrast, VCP746 produced increases in heart rate and renal and mesenteric vascular conductance that were not mediated by A_1_‐receptors. *In vitro* studies confirmed that VCP746 had potent agonist activity at both A_2A_‐ and A_2B_‐receptors.

**Conclusions and Implications:**

These results suggest VCP746 mediates its cardiovascular effects via activation of A_2_ rather than A_1_ adenosine receptors. This has implications for the design of future bitopic ligands that incorporate A_1_ allosteric ligand moieties.

**Abbreviations**
A_1_‐receptor: A_1_ adenosine receptor
Capadenoson: 2‐amino‐6‐[[2‐(4‐chlorophenyl)‐1,3‐thiazol‐4‐yl]methylsulfanyl]‐4‐[4‐(2‐hydroxyethoxy)phenyl]pyridine‐3,5‐dicarbonitrile
CCPA: (2‐chloro‐*N* ^6^‐cyclopentyladenosine)
DPCPX: 1,3‐dipropyl‐8‐cyclopentylxanthine
NECA: 5′‐(*N*‐ethylcarboxamido)adenosine
VCP171: (2‐amino‐4‐(3‐(trifluoromethyl)phenyl)thiophen‐3‐yl)(phenyl)methanone
VCP746: 4‐(5‐amino‐4‐benzoyl‐3‐(3‐(trifluoromethyl)phenyl)thiophen‐2‐yl)‐*N*‐(6‐(9‐((2*R*,3*R*,4*S*,5*R*)‐3,4‐dihydroxy‐5‐(hydroxylmethyl)tetrahydro‐furan‐2‐yl)‐9*H*‐purin‐6‐ylamino)hexyl)benzamide

**What is already known**

- Adenosine is a local mediator that regulates physiological processes via stimulation of the adenosine A_1_‐receptor.
- VCP746 is adenosine linked to an A_1_‐allosteric enhancer (VCP171), reported to be a biased A_1_‐agonist.

**What this study adds**

- VCP746 causes increases in heart rate and vascular conductance that are not mediated by A_1_‐receptors.
- Reporter gene studies confirm that VCP746 is a potent agonist of both A_2A‐_ and A_2B_‐receptors.

**What is the clinical significance**

- The adenosine receptor subtype selectivity of the bitopic ligand VCP746 and its cardiovascular action have been clarified.

## 1 INTRODUCTION

https://www.guidetopharmacology.org/GRAC/LigandDisplayForward?ligandId=2844 is present in all cells in tightly regulated concentrations (Borea, Gessi, Merighi, Vincenzi, & Varani, 2018; Fredholm, Ijzerman, Jacobson, Linden, & Müller, 2011). It is released under a variety of physiological and pathophysiological conditions to facilitate protection and regeneration of tissues (Borea et al., 2018; Fredholm et al., 2011; Müller & Jacobson, 2011). Adenosine acts via four different GPCRs (https://www.guidetopharmacology.org/GRAC/ObjectDisplayForward?objectId=18, https://www.guidetopharmacology.org/GRAC/ObjectDisplayForward?objectId=19, https://www.guidetopharmacology.org/GRAC/ObjectDisplayForward?objectId=20, and https://www.guidetopharmacology.org/GRAC/ObjectDisplayForward?objectId=21) to mediate its physiological effects. A_1_‐ and A_3_‐receptors are Gi/o‐coupled and mediate responses primarily via inhibition of https://www.guidetopharmacology.org/GRAC/FamilyDisplayForward?familyId=257 activity and/or inhibition of neurotransmitter release (Fredholm et al., 2011; Hill, May, Kellam, & Woolard, 2014; Müller & Jacobson, 2011). A_2A_‐ and A_2B_‐receptors are Gs‐coupled and mediate their effects primarily via stimulation of AC activity (Borea et al., 2018; Fredholm et al., 2011; Müller & Jacobson, 2011).

In the heart, adenosine A_1_‐receptor activation results in negative inotropic, chronotropic, and dromotropic effects (Borea et al., 2018; Fenton & Dobson, 2007; Headrick, Peart, Reichelt, & Haseler, 2011). In addition, it has been shown that selective A_2A_ adenosine receptor agonists are potent vasodilators in the rat that reduce BP and induce marked increases in heart rate and plasma https://www.guidetopharmacology.org/GRAC/ObjectDisplayForward?objectId=2413 activity (Alberti et al., 1997). The A_2A_ effect on heart rate appears to be secondary to an increase in reflex sympathetic nervous activity (Alberti et al., 1997). Previous work in conscious rats has also shown that an A_1_‐selective agonist can cause significant decreases in heart rate, a fall in mean arterial pressure and vasoconstriction in both the renal and mesenteric vascular beds, but vasodilatation in the hindquarters (Jolly, March, Kemp, Bennett, & Gardiner, 2008).

A number of selective agonists and antagonists are now available for the A_1_‐receptor (Borea et al., 2018; Fredholm et al., 2011; Müller & Jacobson, 2011). Some A_1_‐receptor‐selective agonists and partial agonists have undergone evaluation for cardiovascular disease indications such as paroxysmal supraventricular tachycardia, atrial fibrillation, angina pectoris and heat failure (Meibom et al., 2017; Müller & Jacobson, 2011). However, the ubiquitous distribution of adenosine receptors in the body can often limit therapeutic application because of the effects of adenosine receptor ligands on the same receptor in a different tissue or cell type (Müller & Jacobson, 2011).

One way in which the activity of endogenous adenosine can be subtly regulated at the level of its target receptor is via drugs that bind to an allosteric site on the receptor protein of interest. These allosteric modulators act to enhance or inhibit the binding of adenosine to its receptor binding site (the orthosteric site) and/or change the resulting functional response (Keov, Sexton, & Christopoulos, 2010; Hill et al., 2014; Göblyös & IJzerman, 2011; Kimatrai‐Salvador, Baraldi, & Romagnoli, 2012; Cooper et al., 2019). VCP171, (2‐amino‐4‐(3‐(trifluoromethyl)phenyl)thiophen‐3‐yl)(phenyl)methanone, has been described as a novel positive allosteric modulator for the adenosine A_1_‐receptor (Aurelio et al., 2009, 2010; Imlach, Bhola, May, Christopoulos, & Christie, 2015; Valant et al., 2010; Vincenzi et al., 2014). Recently, a hybrid molecule (or bitopic ligand) has been described that comprises adenosine attached via a linker to this positive allosteric modulator (Valant et al., 2014). The resulting hybrid ligand (VCP746) has been suggested to exhibit signalling bias towards Gi‐mediated signalling and furthermore was also able to protect against ischaemic damage in cardiomyocytes expressing native A_1_‐receptors (Valant et al., 2014). It did not, however, alter heart rate in an isolated rat atrial preparation (Valant et al., 2014). VCP746 has also been shown to reduce cardiac myocyte hypertrophy and remodelling (Chuo et al., 2016).

**Figure 1:**
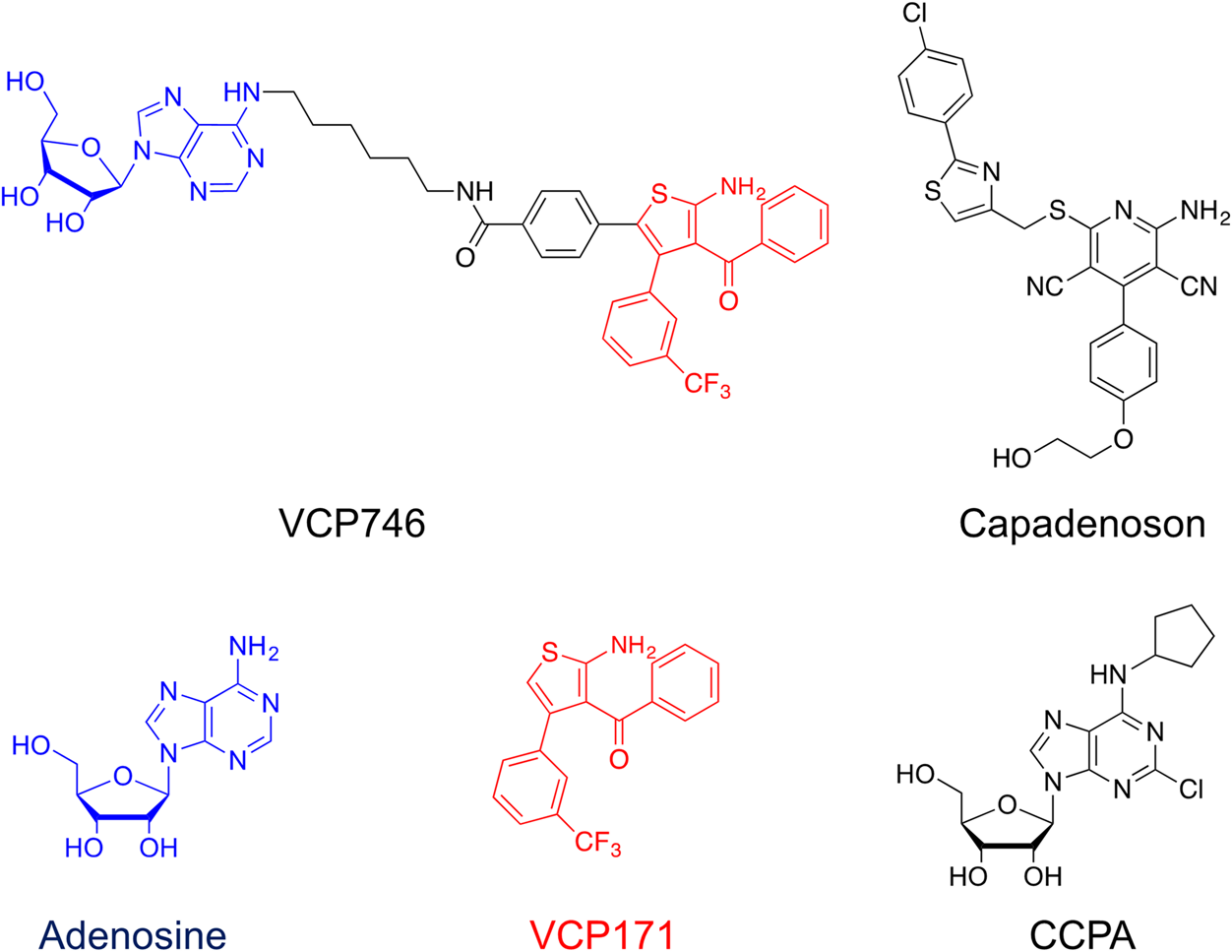
Chemical structures of adenosine, capadenoson, CCPA, VCP171 and VCP746. In the case of VCP746, the adenosine and VCP171 component of the structure are shown in blue and red, respectively

In the present study, we have used a Doppler flow in vivo model for the simultaneous measurement of vascular conductance in three distinct vascular beds, heart rate and BP in conscious freely moving rats, to compare the cardiovascular effects of a full A_1_‐receptor‐selective agonist https://www.guidetopharmacology.org/GRAC/LigandDisplayForward?ligandId=374, a partial agonist capadenoson (Tendera et al., 2012) and VCP746 (Figure 1). This allows us to evaluate the impact of adenosine receptor bitopic ligand VCP746 on cardiovascular responses in an animal model subject to normal autonomic regulation.

## 2 METHODS

### 2.1 Drugs, chemical reagents and other material

Adenosine receptor ligands, adenosine (Cat# A9251) and 5′‐*N*‐ethylcarboxamidoadenosine (https://www.guidetopharmacology.org/GRAC/LigandDisplayForward?ligandId=377; Cat# E2387) were purchased from Sigma‐Aldrich (Gillingham, UK). 1,3‐Dipropyl‐8‐cyclopentylxanthine (https://www.guidetopharmacology.org/GRAC/LigandDisplayForward?ligandId=386; Cat# C101) and 2‐chloro‐*N* ^6^‐cyclopentyladenosine (CCPA; Cat# C7938) were purchased from Tocris Bioscience (Bristol, UK). (2‐Amino‐4‐(3‐(trifluoromethyl)phenyl)thiophen‐3‐yl)(phenyl)methanone (VCP171) and 4‐(5‐amino‐4‐benzoyl‐3‐(3‐(trifluoromethyl)phenyl)thiophen‐2‐yl)‐*N*‐(8‐(9‐((2*R*,3*R*,4*S*,5*R*)‐3,4‐dihydroxy‐5‐(hydroxylmethyl)tetrahydro‐furan‐2‐yl)‐9*H*‐purin‐6‐ylamino)hexyl)benzamide (VCP746) were synthesized as previously described by Aurelio et al. (2009) and Valant et al. (2014), respectively. 2‐Amino‐6‐[[2‐(4‐chlorophenyl)‐1,3‐thiazol‐4‐yl]methylsulfanyl]‐4‐[4‐(2‐hydroxyethoxy)phenyl]pyridine‐3,5‐dicarbonitrile (capadenoson) was purchased from Haoyuan Chemexpress (Cat# HY‐14917; Shanghai, China).

Fentanyl citrate was purchased from Jansen‐Cilac Ltd, UK. Medetomidine (Domitor), meloxicam, and atipamezole hydrochloride (Antisedan) were purchased from Pfizer, UK. Buprenorphine (Vetergesic) and pentobarbitone (Euthatal) were purchased from Alstoe Animal Health, UK. Tween and propylene glycol were purchased from Sigma‐Aldrich, UK.

### 2.2 CRE‐SPAP gene transcription assay for adenosine A_2A_‐ and A_2B_‐receptor agonist activity in CHO‐K1 cells

CHO‐K1 cells (RRID‐https://web.expasy.org/cellosaurus/CVCL_0214) expressing a https://www.guidetopharmacology.org/GRAC/LigandDisplayForward?ligandId=2352 response element (CRE) regulated secreted placental alkaline phosphate (SPAP) reporter gene and the human adenosine A_2A_‐receptor or human adenosine A_2B_‐receptor were generated and grown to confluence in clear 96‐well plates as described previously (Stoddart, Vernall, Briddon, Kellam, & Hill, 2015). On the day prior to analysis, normal growth medium was removed and replaced with serum‐free medium (SFM; DMEM/F12 supplemented with 2‐mM l‐glutamine). On the day of the experiment, fresh SFM was added to the cells with increasing concentrations of the required test compounds. CRE‐SPAP cells were then incubated for 5 hr at 37°C in humidified air containing 5% CO_2_. After 5‐hr incubation, all medium was removed from the cells and replaced with 40 μl of SFM and incubated for a further 1 hr. The plates were then incubated at 65°C for 30 min to destroy the endogenous alkaline phosphatases. After cooling the plates to room temperature, 100 μl of 5‐mM 4‐nitrophenyl phosphate in a diethanolamine‐containing buffer (10% (v/v) diethanolamine, 280‐mM NaCl, 500‐mM MgCl_2_, pH 9.85) was then added to each well. Plates were incubated for varying times depending on the cell line (CHO‐A_2A_, 25 min at 37°C; CHO‐A_2B_, overnight at room temperature) and then the absorbance at 405 nm was measured using a Dynex MRX plate reader (Chelmsford, MA, USA). Five independent experiments were conducted using quadruplicate repeats to ensure the reliability of single values.

### 2.3 Animals and surgery

Adult male Sprague–Dawley rats weighing 350 to 450 g were purchased from Charles River Laboratories, UK. Animals were pair housed in standard, individually ventilated cages prepared with bedding material and enrichment. Cages were held in a temperature‐controlled (21–23°C) environment with a 12‐hr light–dark cycle (lights on at 6:00 a.m.), and animals received access to food (18% Protein Rodent Diet; Envigo, Madison WI, USA) and water ad libitum for at least 7 days before any surgical intervention. All procedures were approved by the Animal Welfare and Ethical Review Body (University of Nottingham; which has representation from NC3Rs) and performed in keeping with the Animals (Scientific Procedures) Act (1986), under UK Home Office approved Project Licence and Personal License authority (PPL 40/3680). Forty five rats were used for this study, and results are recorded in accordance with the ARRIVE guidelines for reporting experiments involving animals (McGrath, Drummond, McLachlan, Kilkenny, & Wainwright, 2010). Animal studies are reported in compliance with the ARRIVE guidelines (Kilkenny et al., 2010) and the editorial on reporting animal studies (McGrath & Lilley, 2015), with the recommendations made by the *British Journal of Pharmacology.*

### 2.4 Doppler flow probe implantation

Surgery was performed under general anaesthesia (https://www.guidetopharmacology.org/GRAC/LigandDisplayForward?ligandId=1626 and medetomidine, 300 μg·kg^−1^ each, i.p., supplemented as required), with reversal of anaesthesia and postoperative analgesia provided by atipamezole hydrochloride (1 mg·kg^−1^, s.c.) and https://www.guidetopharmacology.org/GRAC/LigandDisplayForward?ligandId=1670e (30 μg·kg^−1^, s.c.). A second dose of buprenorphine (15 μg·kg^−1^, s.c.) was given as an analgesic 4 hr after surgery.

A midline incision was performed and miniature pulsed Doppler flow probes were sutured around the left renal and superior mesenteric arteries and the descending abdominal aorta (providing blood to the hindquarters) to monitor vascular conductance (VC; Carter, Fretwell, & Woolard, 2017). The probe wires were secured to the left abdominal wall and tunnelled via the left flank to the posterior of the neck. The wires were then secured with suture and sterile tape to the nape of the neck. Following surgery, animals were returned to home cages with access to food and water and received analgesia (meloxicam 1 mg·kg^−1^·day^−1^) for 3 days.

### 2.5 Catheter implantation

Catheter implantation commenced at least 10 days after the Doppler flow probe implantation surgery and after a satisfactory inspection from the Named Veterinary Surgeon. Under anaesthesia (as described above) catheters filled with heparinised saline (15 U·ml^−1^) were implanted into the distal abdominal aorta, via the ventral caudal artery (for the measurement of arterial BP and heart rate) and implanted into the right jugular vein (for drug administration; Carter et al., 2017). Three separate intravenous catheters were placed in the jugular vein to enable independent administration of different substances. At this stage, the probe wires were soldered into a miniature plug (Omnetic Connector Corporation, USA), which was mounted onto a custom‐designed harness worn by the rat. The catheters emerged from the same point as the probe wires and were fed through a protective spring secured to the harness and attached to a counterbalanced pivot system. Reversal of anaesthetic and analgesia was administered (as described above) and the animals were single housed with free access to food and water. The arterial catheter was connected to a fluid‐filled swivel for overnight infusion of heparinised (15 U·ml^−1^) saline to maintain potency.

Experiments began 24 hr after surgery for catheter implantation, with animals fully conscious and unrestrained in home cages and with free access to food and water.

### 2.6 Cardiovascular recordings

Haemodynamic variables (heart rate, arterial BP, renal, mesenteric, and hindquarters Doppler shifts) were recorded using a customized, computer‐based system (IdeeQ; Maastricht Instruments, Maastricht, The Netherlands) that connected a transducer amplifier (13‐4615‐50; Gould, Cleveland, OH, USA), a Doppler flowmeter (Crystal Biotech, Holliston, MA, USA), and a VF‐1 mainframe (pulse repetition frequency 125 kHz) fitted with high‐velocity (HVPD‐20) modules. Raw data were sampled by IdeeQ every 2 ms, averaged, and stored to disc every cardiac cycle. Changes in renal VC (RVC), mesenteric VC (MVC), and hindquarter VC (HVC), in the renal, mesenteric, and hindquarter vascular beds, respectively, were calculated from the changes in mean arterial pressure (MAP) and Doppler shift. At the end of each experiment, rats were killed by a schedule one procedure, with Euthatal (60–80 mg, i.p.) and exsanguination.

### 2.7 Experimental protocol

Experiments were run in six studies, each lasting 3 days; within each study was a contemporaneous vehicle control (5% propylene glycol, 2% Tween 80 in sterile saline). Power calculations were performed to estimate appropriate group sizes, and experiments were generally run with treatment groups of six to eight rats. Animals were randomly allocated to antagonist or vehicle on day 1 and the alternative treatment then given on day 3.

#### 2.7.1 Study 1

Eight animals were used to assess the cardiovascular responses to CCPA in the presence or absence of DPCPX. After a period of baseline recordings, rats were randomized into two groups. **Group 1** received vehicle intravenous bolus (0.1 ml provided over 5 s) on day 1 and a DPCPX (0.1 mg·kg^−1^) intravenous bolus on day 3. **Group 2** received DPCPX (0.1 mg·kg^−1^, 0.1‐ml bolus, i.v.) on day 1 and a vehicle (0.1‐ml bolus, i.v.) on day 3. Approximately 10 min after the initial bolus of vehicle or DPCPX, all groups received intravenous infusions (0.1 ml·min^−1^) of CCPA (120 [low], 400 [mid], and 1,200 [high] ng·kg^−1^·min^−1^) on both day 1 and day 3. Each dose was infused for 3 min. Haemodynamic recordings were made for a further 4 hr following the completion of the CCPA intravenous infusion period.

#### 2.7.2 Study 2

Seven animals were used to measure the cardiovascular responses to capadenoson in the presence or absence of DPCPX. Following a period of baseline, rats were randomized into two groups. **Group 1** received vehicle (0.1 ml bolus, i.v.) on day 1 and DPCPX (0.1 mg·kg^−1^ bolus, i.v.) on day 3. **Group 2** received a single 0.1 ml bolus of DPCPX (0.1 mg·kg^−1^, i.v.) on day 1 and vehicle (bolus, 0.1 ml) on day 3. After 10 min, all groups received intravenous infusions (0.1 ml·min^−1^) of capadenoson (30 [low], 100 [mid], and 300 [high] μg·kg^−1^·min^−1^) on day 1 and day 3. Each dose of capadenoson was given as a 3‐min infusion. Cardiovascular recordings were continued for a further 4 hr after administration of capadenoson.

#### 2.7.3 Study 3

Eight animals were used to assess the cardiovascular responses to VCP746 (Valant et al., 2014) in the presence or absence of DPCPX. Following a period of baseline, rats were randomized into two groups. **Group 1** received vehicle (0.1 ml bolus, i.v.) on day 1 and DPCPX (0.1 mg·kg^−1^ bolus, i.v.) on day 3. **Group 2** received a single 0.1 ml bolus of DPCPX (0.1 mg·kg^−1^, i.v.) on day 1 and vehicle (bolus, 0.1 ml) on day 3. Approximately 10 min after the initial bolus, all groups received intravenous infusions (0.1 ml·min^−1^) of VCP746 (6 [low], 20 [mid], and 60 [high] μg·kg^−1^·min^−1^) on day 1 and day 3. Each dose of VCP746 was given as a 3‐min infusion. Cardiovascular recordings were continued for a further 4 hr after administration of the VCP746.

#### 2.7.4 Study 4

Eight animals were used to assess the cardiovascular responses to adenosine in the presence or absence of DPCPX. Following a period of baseline, rats were randomized into two groups. **Group 1** received vehicle (0.1 ml bolus, i.v.) on day 1 and DPCPX (0.1 mg·kg^−1^ bolus, i.v.) on day 3. **Group 2** received a single 0.1 ml bolus of DPCPX (0.1 mg·kg^−1^, i.v.) on day 1 and vehicle (bolus, 0.1 ml) on day 3. After 10 min, all groups received intravenous infusions (0.1 ml·min^−1^) of adenosine (30 [low], 100 [mid], and 300 [high] μg·kg^−1^·min^−1^) on day 1 and day 3. Each dose of adenosine was given as a 3‐min infusion. Cardiovascular recordings were continued for a further 4 hr after administration of the adenosine.

#### 2.7.5 Study 5

Seven animals were used to assess the cardiovascular responses to VCP171 (Aurelio et al., 2009) in the presence or absence of DPCPX. Following a period of baseline, rats were randomized into two groups. **Group 1** received vehicle (0.1 ml bolus, i.v.) on day 1 and DPCPX (0.1 mg·kg^−1^ bolus, i.v.) on day 3. **Group 2** received a single 0.1 ml bolus of DPCPX (0.1 mg·kg^−1^, i.v.) on day 1 and vehicle (bolus, 0.1 ml) on day 3. After 10 min, all groups received intravenous infusions (0.1 ml·min^−1^) of VCP171 (2.6 [low], 8.5 [mid], and 25.6 [high] μg·kg^−1^·min^−1^) on day 1 and day 3. Each dose of VCP171 was given as a 3 min infusion. Cardiovascular recordings were continued for a further 4 hr after administration of the adenosine.

#### 2.7.6 Study 6

Seven animals were used to assess the cardiovascular responses to adenosine in the presence or absence of VCP171. Following a period of baseline, rats were randomized into two groups. **Group 1** received a 14‐min infusion of vehicle (0.1 ml·min^−1^, i.v.) on day 1 and a 14‐min infusion of VCP171 (25.6 μg·kg^−1^·min^−1^, i.v.) on day 3. **Group 2** received a 14‐min infusion of VCP171 (25.6 μg·kg^−1^·min^−1^, i.v.) on day 1 and a vehicle infusion (0.1 ml·min^−1^) on day 3. After 5 min of the VCP171 or vehicle infusion, all groups received intravenous infusions (0.1 ml·min^−1^) of adenosine (30 [low], 100 [mid], and 300 [high] μg·kg^−1^·min^−1^) on day 1 and day 3. Each dose of adenosine was given for 3 min. Cardiovascular recordings were continued for a further 4 hr after administration of the adenosine.

### 2.8 Data analysis

**Figure 2:**
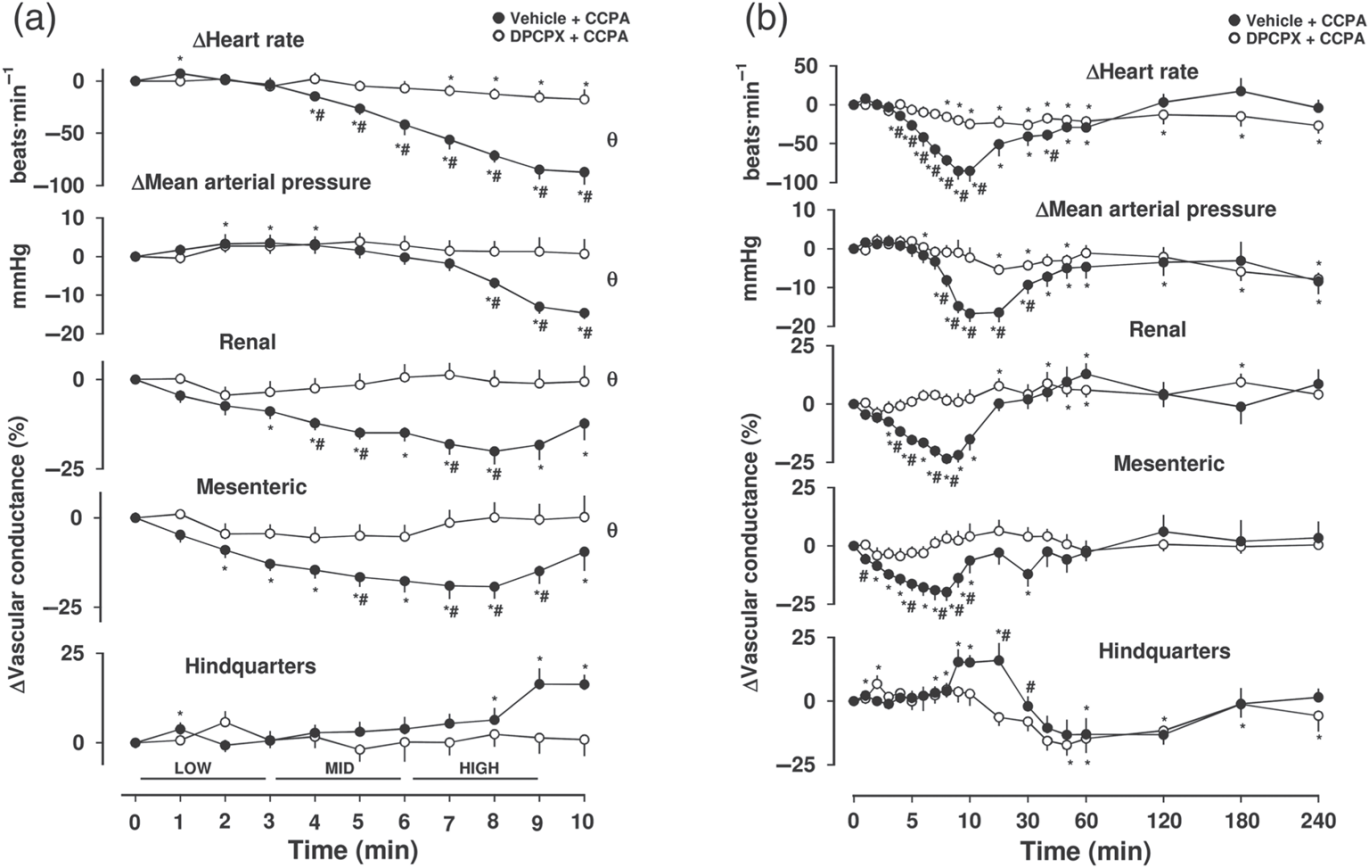
Cardiovascular responses to CCPA in the presence or absence of DPCPX, in conscious freely moving rats. Rats were dosed with either DPCPX (0.1 ml bolus dose of 0.1 mg·kg^−1^, i.v., *n* = 8) or vehicle (0.1 ml bolus dose of 5% propylene glycol, 2% Tween 80 in sterile saline, *n* = 8), as described under Section 2. Approximately 10 min later, all animals received an infusion of CCPA (120 [low], 400 [mid] and 1,200 [high] ng·kg^−1^·min^−1^; each dose infused over 3 min). Time course is shown during (a) the treatment period and (b) over the extended 4‐hr recording period. Data points are mean and vertical bars represent SEM. ^*^ *P* < .05 versus baseline (Friedman's test). *θ* significant difference between groups comparison (*P* < .05; Wilcoxon signed rank test, 0–5 min) based on integrated area under or above curve analysis. A Wilcoxon test was also conducted between treated and vehicle controls at each time point (^#^ *P* < .05)

All in vivo data were collected and analysed using IdeeQ software 2.5 (Maastricht Instruments, Maastricht University, NL). For all experiments, time‐averaged data are shown as changes from baseline [heart rate (HR) (beats·min^−1^); MAP (mmHg); VC (%)]. Statistical analysis used BIO‐medical software 3.4 (Medical Physics, University of Nottingham, UK). Statistical comparisons between groups of animals were performed on the integrated changes over specified time periods. A Friedman test, which is a nonparametric, repeated‐measures ANOVA was used for within‐group comparisons and a Wilcoxon rank‐sum test for integrated area under or above curve analysis was used for comparisons between groups. A Wilcoxon test was also performed for comparisons between groups at a specific time point. Vascular conductances were calculated from the MAP and Doppler shift (flow) data. It is worth noting that the Friedman test is a non‐parametric statistical test which involves ranking each row and then comparing the values of ranked columns, to detect differences in treatments across multiple time points. Variability within the row can therefore affect the level of significance. Thus, certain points of significance over a period of 10 min may not always retain significant when analysed over the extended 240 min (e.g., when comparing (a) and (b) in Figures 2, 5, 6, 7, 8). For this reason, statistical analysis was performed over both time periods.

Statistical analysis of the combine data for vehicle (*n* = 38) and DPCPX alone (*n* = 38) from all studies was also analysed by Wilcoxon signed rank test for differences between groups at a specific time point and Friedman's test to test for within‐group differences from the time zero point using Prism 7.0 (GraphPad Software, San Diego, CA, USA). Prism 7.0 was used instead of BIO‐medical software due to the large number of data sets used.

Concentration–response curves for agonist‐mediated CRE‐SPAP gene expression were fitted using non‐linear regression (Prism 7.0, GraphPad Software, San Diego, CA, USA) to the following equation:

$$\text{Response}=\frac{E_{\max}\times \left[A\right]}{\left[A\right]+{\mathrm{EC}}_{50}},$$

where *E*_max_ is the maximal response, [*A*] is the concentration of agonist, and the EC_50_ is the molar concentration of agonist required to generate 50% of the *E*_max_. Statistical analysis of concentration–response data was analysed by one‐way ANOVA using Prism 7.0. For all statistical analysis, a value of *P* < .05 was considered significant. The data and statistical analysis comply with the recommendations of the *British Journal of Pharmacology* on experimental design and analysis in pharmacology (Curtis et al., 2018).

### 2.9 Nomenclature of targets and ligands

Key protein targets and ligands in this article are hyperlinked to corresponding entries in http://www.guidetopharmacology.org, the common portal for data from the IUPHAR/BPS Guide to PHARMACOLOGY (Harding et al., 2018) and are permanently archived in the Concise Guide to PHARMACOLOGY 2017/18 (Alexander, Christopoulos, et al., 2017; Alexander, Fabbro, et al., 2017).

## 3 RESULTS

Baseline cardiovascular variables before the administration of 1,3‐dipropyl‐8‐cyclopentylxanthine (DPCPX), adenosine receptor agonists and their corresponding vehicles controls are shown in Table 1.

**Table 1:** Cardiovascular variables prior to administration of adenosine agonists and antagonists

	Study 1		Study 2		Study 3		Study 4		Study 5		Study 6	
	Vehicle	DPCPX	Vehicle	DPCPX	Vehicle	DPCPX	Vehicle	DPCPX	Vehicle	DPCPX	Vehicle	VCP171
Baseline: Time 0												
Heart rate (beats·min^−1^)	352 ± 13	343 ± 6	340 ± 7	359 ± 14	344 ± 10	355 ± 8*	351 ± 9	351 ± 10	361 ± 16	362 ± 12	327 ± 6	354 ± 12
Mean BP (mmHg)	106 ± 6	101 ± 5	104 ± 4	99 ± 2	102 ± 2	101 ± 2	98 ± 2	102 ± 4	103 ± 2	106 ± 4	106 ± 4	109 ± 3
Renal VC (U)	73 ± 11	70 ± 8	73 ± 9	66 ± 8	79 ± 7	70 ± 14	72 ± 6	79 ± 10	77 ± 6	83 ± 6	87 ± 7	92 ± 8
Mesenteric VC (U)	78 ± 9	80 ± 9	99 ± 8	99 ± 6	121 ± 9	103 ± 8	100 ± 12	88 ± 10	100 ± 4	98 ± 6	94 ± 12	87 ± 10
Hindquarters VC (U)	63 ± 8	69 ± 16	47 ± 6	57 ± 9	49 ± 2	51 ± 4	59 ± 5	59 ± 12	46 ± 3	48 ± 6	44 ± 3	46 ± 4
Prior to infusion: Time 6–10 min												
Heart rate (beats·min^−1^)	344 ± 11	347 ± 8	343 ± 9	366 ± 10	343 ± 12	357 ± 8	343 ± 9	351 ± 13	357 ± 17	355 ± 16	320 ± 6	345 ± 13
Mean BP (mmHg)	107 ± 5	103 ± 4	105 ± 4	104 ± 2	101 ± 3	103 ± 2	99 ± 2	107 ± 2	103 ± 2	107 ± 4	111 ± 4	111 ± 3
Renal VC (U)	69 ± 10	64 ± 7	74 ± 9	63 ± 8	80 ± 5	69 ± 7	80 ± 8	76 ± 6	76 ± 6	79 ± 5	82 ± 8	97 ± 10
Mesenteric VC (U)	83 ± 5	77 ± 6	94 ± 8	92 ± 6	121 ± 10	99 ± 10	96 ± 12	74 ± 10	96 ± 4	85 ± 5	80 ± 12	83 ± 10
Hindquarters VC (U)	58 ± 5	65 ± 13	44 ± 6	55 ± 8	49 ± 3	54 ± 2	59 ± 5	55 ± 10	48 ± 4	45 ± 3	44 ± 5	50 ± 5

*Note.* Values are mean ± *SE.* Units of vascular conductance (VC) are kHz. mmHg^−1^ × 10^3^. *N* = 7–8 per group.

Abbreviations: U, units; VC, vascular conductance.

* *P* < .05 versus corresponding vehicle group.

### 3.1 Effect of 2‐chloro‐*N* ^6^‐cyclopentyladenosine (CCPA)

Intravenous infusion of increasing concentrations of the adenosine A_1_‐receptor‐selective agonist CCPA (120, 400 and 1,200 ng·kg^−1^·min^−1^; 3‐min infusions of each dose) produced a significant dose‐dependent decrease (*P* < .05) in heart rate and MAP (Figure 2a,b). This was accompanied by vasoconstriction in both the renal and mesenteric vascular beds, but a small vasodilatation in the hindquarters (*P* < .05; Figure 2a,b). The increase in VC in the hindquarters (monitored via the Doppler probe on the descending abdominal aorta) developed more slowly than the vasoconstriction observed in the renal and mesenteric vascular beds and reached a peak at 10 min which, unlike the effects on HR and renal or mesenteric VC, was maintained for a further 10 min (Figure 2b). The fall in MAP also developed more slowly and was sustained until the 20‐min time point (Figure 2b). A role for adenosine A_1_‐receptors in these cardiovascular responses was confirmed by pre‐treating animals with a bolus intravenous injection of the A_1_‐selective antagonist DPCPX (0.1 mg·kg^−1^, i.v.) prior to the addition of CCPA. DPCPX pretreatment significantly attenuated (*P* < .05) the CCPA‐induced effects on HR, MAP and VC in the renal and mesenteric vascular beds (Figure 2a,b). The effect of DPCPX on the effect of CCPA on HVC did not reach significance (Figure 2a,b).

**Figure 3:**
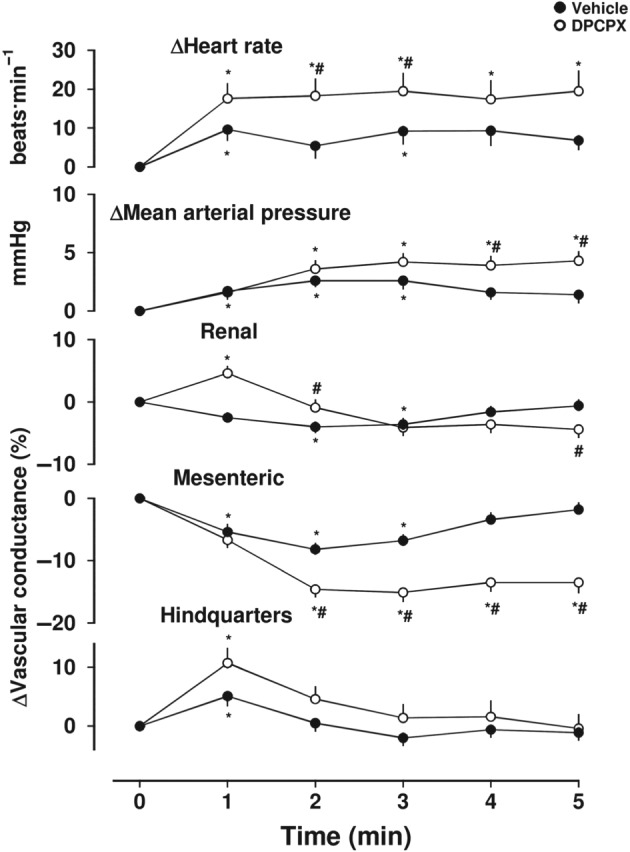
Cardiovascular responses to DPCPX, in conscious freely moving rats. Rats were dosed with either DPCPX (0.1 ml bolus dose of 0.1 mg·kg^−1^ i.v., *n* = 38) or vehicle (0.1 ml bolus dose of 5% propylene glycol, 2% Tween 80 in sterile saline, *n* = 38), as described under Section 2. The time course shows responses over the 5‐min period, post‐dosing. Data points are mean and vertical bars represent SEM. ^*^ *P* < .05 versus baseline (Friedman's test). A Wilcoxon signed rank test was also conducted between treated and vehicle controls at each time point (^#^ *P* < .05)

The effect of DPCPX alone was evaluated for 5 min (relative to a vehicle control) prior to agonist administration in all five of the studies involving DPCPX described under Section 2. Figure 3 shows the combined data for DPCPX and vehicle from all of the studies. Administration of DPCPX produced a small increase in HR and MAP consistent with antagonism of a small contribution to basal activity from an A_1_‐receptor‐mediated response to endogenous adenosine (Figure 3). There was also a small direct effect of DPCPX on MVC (Figure 3). A similar effect on MVC has been previously reported by Jolly et al. (2008) who also found that mesenteric vasoconstrictor effects of DPCPX were reduced by losartan suggesting that it may be, at least in part, secondary to antagonism of A_1_‐receptor‐mediated effects of endogenous adenosine on renin release (Jackson, 1991; Tagawa & Vander, 1970).

### 3.2 Effect of the A_1_‐receptor‐selective partial agonist capadenoson

**Figure 4:**
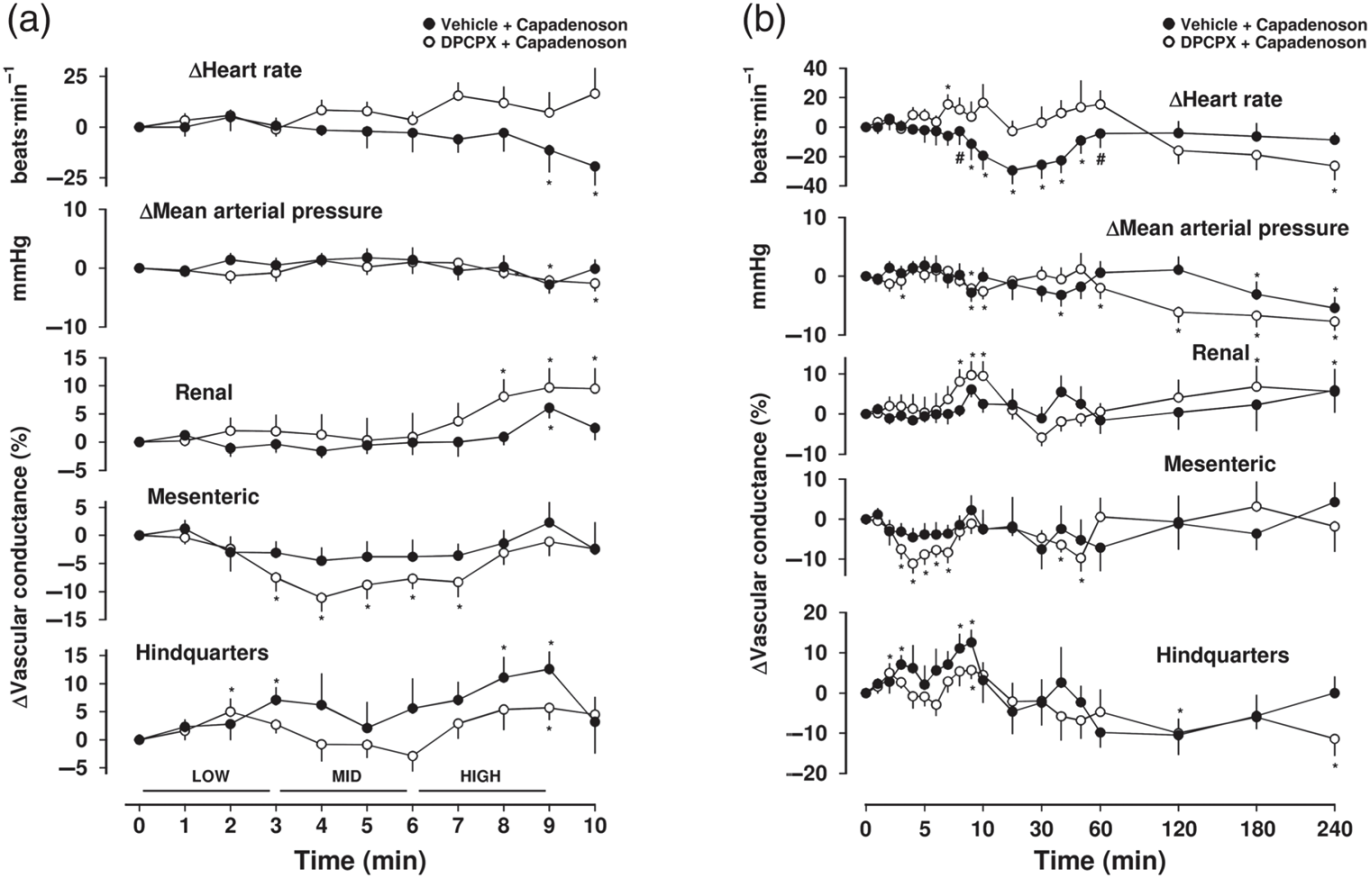
Cardiovascular responses to capadenoson in the presence or absence of DPCPX, in conscious freely moving rats. Rats were dosed with either DPCPX (0.1 ml bolus dose of 0.1 mg·kg^−1^ i.v., *n* = 7) or vehicle (0.1 ml bolus dose of 5% propylene glycol, 2% Tween 80 in sterile saline, *n* = 7), as described under Section 2. Approximately 10 min later, all animals received an infusion of capadenoson (30 [low], 100 [mid], and 300 [high] μg·kg^−1^·min^−1^; each dose infused over 3 min). Time course is shown during (a) the treatment period and (b) over the extended 4‐hr recording period. Data points are mean and vertical bars represent SEM. ^*^ *P* < .05 versus baseline (Friedman's test). A Wilcoxon signed rank test was also conducted between treated and vehicle controls at each time point (^#^ *P* < .05)

Capadenoson is a selective A_1_‐receptor partial agonist (Albrecht‐Kupper, Leineweber, & Nell, 2012) that may have clinical utility in conditions such as angina (Tendera et al., 2012). Intravenous infusion of increasing concentrations of capadenoson (30, 100 and 300 μg·kg^−1^min^−1^; 3‐min infusions of each dose) produced a significant dose‐dependent decrease (*P* < .05) in HR that reached a peak at 20 min (Figure 4a,b). This was not accompanied by any significant changes in MAP or renal and mesenteric VC (Figure 4a,b). There was, however, a small vasodilatation in the hindquarters (Figure 4). Pretreatment with DPCPX prevented the A_1_‐receptor‐mediated decrease in HR caused by capadenoson and revealed a small increase in RVC and a vasoconstriction in the mesenteric vascular bed (Figure 4a,b).

### 3.3 Effect of the bitopic A_1_‐receptor ligand VCP746 on cardiovascular responses in the rat

**Figure 5:**
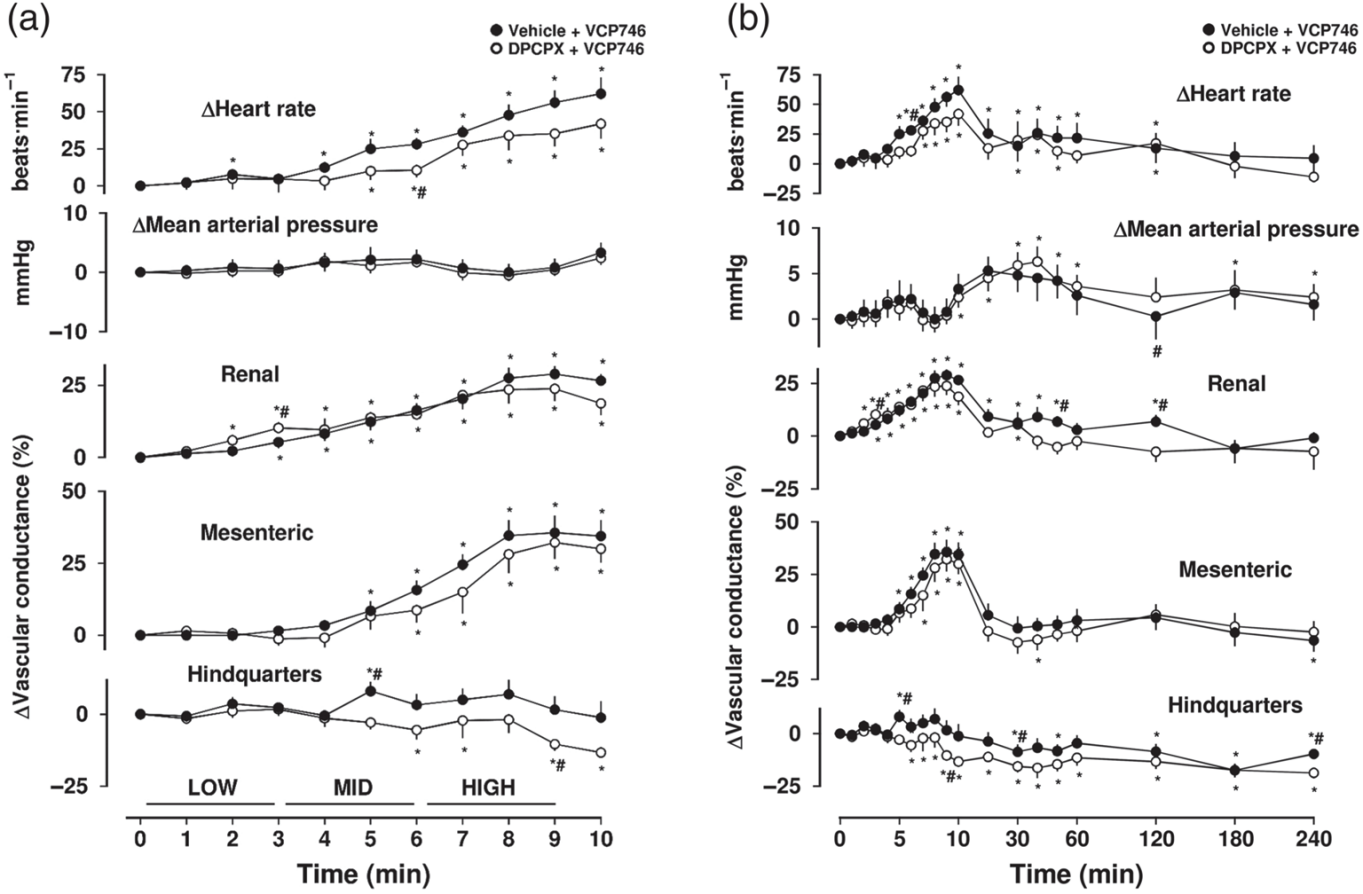
Cardiovascular responses to VCP746 in the presence or absence of DPCPX, in conscious freely moving rats. Rats were dosed with either DPCPX (0.1 ml bolus dose of 0.1 mg·kg^−1^ i.v., *n* = 8) or vehicle (0.1 ml bolus dose of 5% propylene glycol, 2% Tween 80 in sterile saline, *n* = 8), as described under Section 2. Approximately 10 min later, all animals received an infusion of VCP746 (6 [low], 20 [mid], and 60 [high] μg·kg^−1^·min^−1^; each dose infused over 3 min). Time course is shown during (a) the treatment period and (b) over the extended 4‐hr recording period. Data points are mean and vertical bars represent SEM. ^*^ *P* < .05 versus baseline (Friedman's test). A Wilcoxon signed rank test was also conducted between treated and vehicle controls at each time point (^#^ *P* < .05)

VCP746 is a hybrid molecule containing the adenosine moiety linked to the positive A_1_‐receptor allosteric modulator VCP171 (Figure 1). It has been previously reported to act as a biased A_1_‐receptor agonist that has no direct negative chronotropic effect in isolated rat atria (Valant et al., 2014). Application of VCP746 (6, 20 and 60 μg·kg^−1^min^−1^, i.v.; 3‐min infusion), however, produced a significant (*P* < .05) increase in HR in conscious freely moving rats that occurred in parallel with marked increases in renal and mesenteric vascular conductance (Figure 5a,b). These effects of VCP746 were not significantly antagonized by the A_1_‐selective antagonist DPCPX (Figure 5a,b).

### 3.4 Effect of adenosine, VCP171 and both in combination

**Figure 6:**
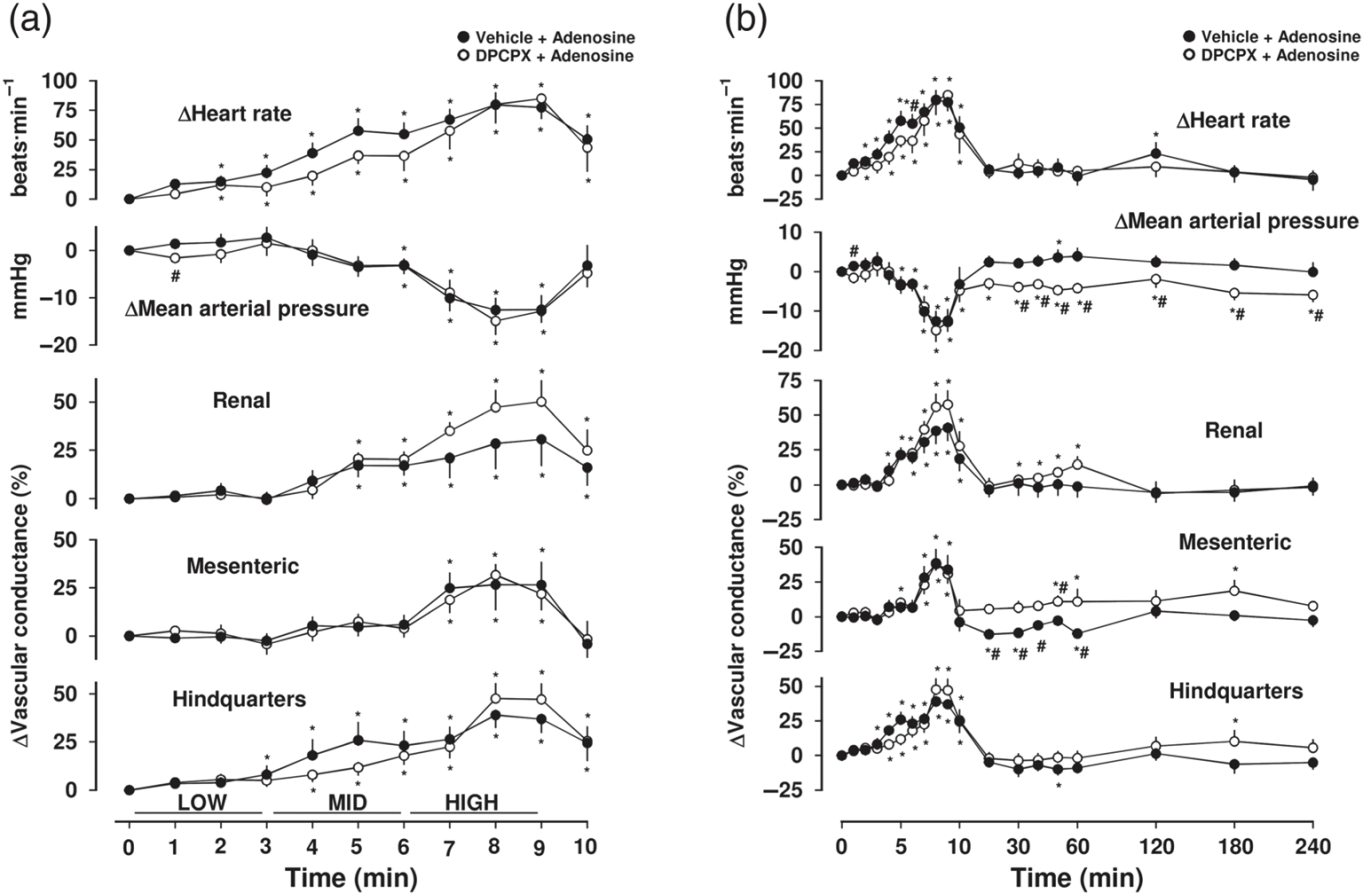
Cardiovascular responses to adenosine in the presence or absence of DPCPX, in conscious freely moving rats. Rats were dosed with either DPCPX (0.1‐ml bolus dose of 0.1 mg·kg^−1^ i.v., *n* = 8) or vehicle (0.1 ml bolus dose of 5% propylene glycol, 2% Tween 80 in sterile saline, *n* = 8), as described under Section 2. Approximately 10 min later, all animals received an infusion of adenosine (30 [low], 100 [mid], and 300 [high] μg·kg^−1^·min^−1^; each dose infused over 3 min). Time course is shown during (a) the treatment period and (b) over the extended 4‐hr recording period. Data points are mean and vertical bars represent SEM. ^*^ *P* < .05 versus baseline (Friedman's test). A Wilcoxon signed rank test was also conducted between treated and vehicle controls at each time point (^#^ *P* < .05)

To investigate the extent to which the cardiovascular responses to VCP746 observed above are due to the component molecules (adenosine and VCP171) contained within VCP746, we have studied their effect on the cardiovascular system alone and in combination. The endogenous ligand adenosine (30, 100 and 300 μg·kg^−1^·min^−1^; 3‐min intravenous infusion) produced a similar cardiovascular profile, in terms of a significant (*P* < .05) increase in HR, and associated increases in renal and mesenteric VC, to that observed with VCP746 (Figure 6a,b). In addition, adenosine also produced a significant fall in MAP (*P* < .05) and a large increase in HVC (*P* < .05; Figure 6a,b). None of the cardiovascular responses to adenosine were prevented by pretreatment with DPCPX. Following infusion of adenosine (Figure 6b), MAP returned to normal levels 60 min after cessation of the adenosine infusion. However, in DPCPX‐treated animals MAP remained slightly lower in the DPCPX‐treated group than in the vehicle controls (Figure 6b). It should be noted, however, that this effect of DPCPX on MAP was only a small and a significant effect of DPCPX treatment was not observed following similar protocols using VCP746, capadenoson or CCPA in place of adenosine (Figures 2, 4, and 5).

**Figure 7:**
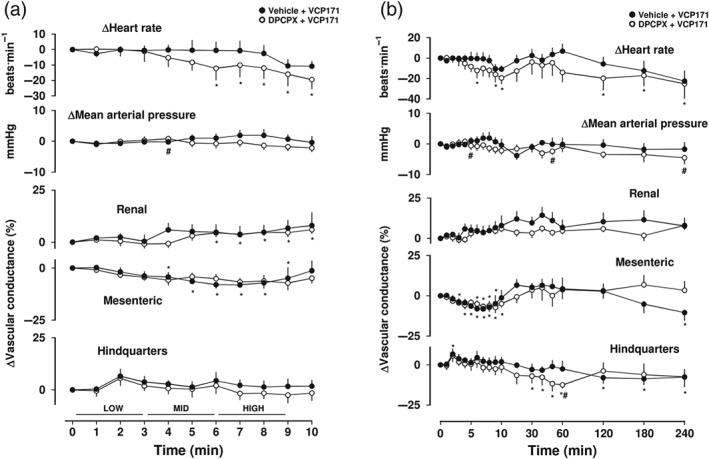
Cardiovascular responses to VCP171 in the presence or absence of DPCPX, in conscious freely moving rats. Rats were dosed with either DPCPX (0.1 ml bolus dose of 0.1 mg·kg^−1^, i.v., *n* = 7) or vehicle (0.1 ml bolus dose of 5% propylene glycol, 2% Tween 80 in sterile saline, *n* = 7), as described under Section 2. Approximately 10 min later, all animals received an infusion of VCP171 (2.6 [low], 8.5 [mid], and 25.6 [high] μg·kg^−1^·min^−1^; each dose infused over 3 min). Time course is shown during (a) the treatment period and (b) over the extended 4‐hr recording period. Data points are mean and vertical bars represent SEM. ^*^ *P* < .05 versus baseline (Friedman's test). A Wilcoxon signed rank test was also conducted between treated and vehicle controls at each time point (^#^ *P* < .05)

The effect of VCP171 alone was investigated at equivalent equimolar doses to those at which the parent hybrid molecule VCP746 elicited significant effects on heart rate and renal/mesenteric VC. The doses chosen were based on the fraction of the MW of VCP746 that was contributed by the VCP171 component (Figure 1). Infusion of VCP171 (2.6, 8.5 and 25.6 μg·kg^−1^·min^−1^; 3‐min intravenous infusions) produced a small decrease in VC in the mesenteric vascular bed (*P* < .05) but no consistent effect on heart rate or renal and hindquarters VC (Figure 7a,b). The reduction in mesenteric VC was not antagonized by DPCPX (Figure 7a,b).

**Figure 8:**
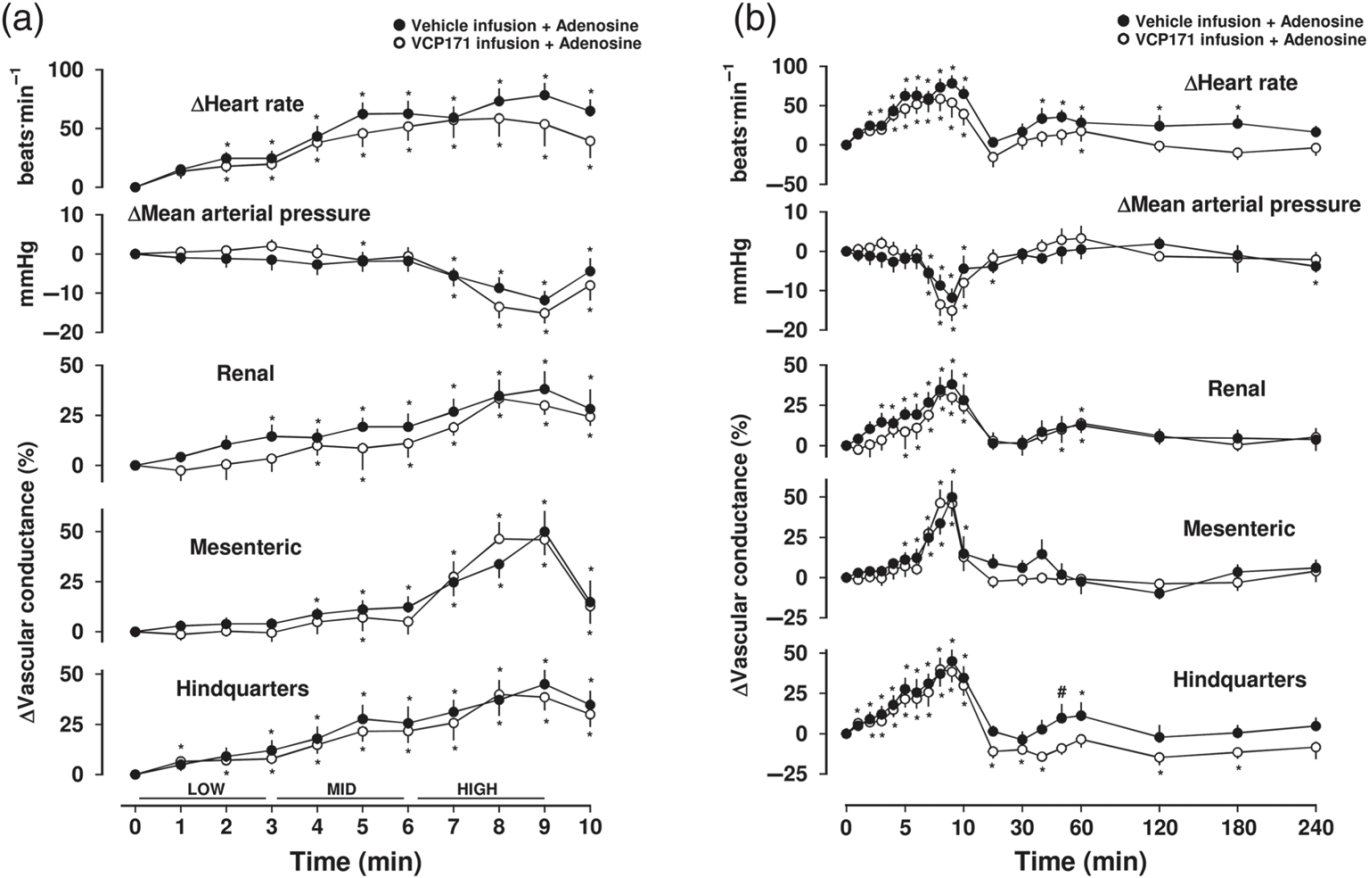
Cardiovascular responses to adenosine in the presence or absence of VCP171, in conscious freely moving rats. Rats were dosed with either VCP171 (25.6 μg·kg^−1^·min^−1^, i.v. for 14 min, *n* = 7) or vehicle (0.1 ml·min^−1^ dose over 14 min of 5% propylene glycol, 2% Tween 80 in sterile saline, *n* = 7), as described under Section 2. Approximately 5 min into the infusion, all animals received an infusion of adenosine (30 [low], 100 [mid], and 300 [high] μg·kg^−1^·min^−1^; each dose infused over 3 min). Time course is shown during (a) the treatment period and (b) over the extended 4‐hr recording period. Data points are mean and vertical bars represent SEM. ^*^ *P* < .05 versus baseline (Friedman's test). A Wilcoxon signed rank test was also conducted between treated and vehicle controls at each time point (^#^ *P* < .05)

In combination, adenosine and VCP171 produced essentially similar cardiovascular responses to those obtained with adenosine alone (Figure 8a,b). In the presence of VCP171, there was a hint that the increase in heart rate produced by adenosine was somewhat blunted, perhaps consistent with an enhanced contribution of a minor A_1_‐receptor‐mediated bradycardia (Figure 8a,b).

### 3.5 Agonist effect of VCP746 on adenosine A_2A_‐ and A_2B_‐receptor‐mediated CRE‐SPAP responses

**Figure 9:**
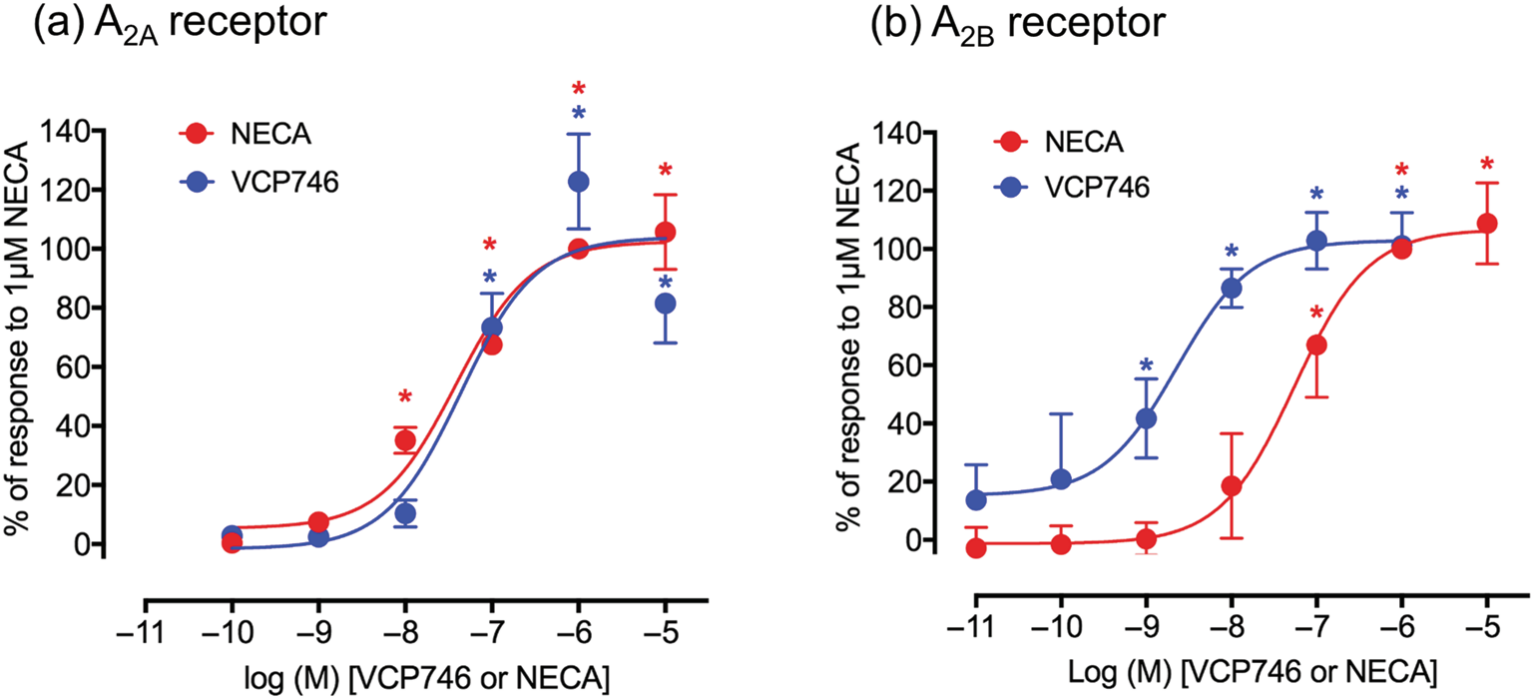
Pharmacological evaluation of agonist‐mediated CRE‐SPAP gene transcription responses to VCP746 and NECA in CHO‐K1 cells expressing a CRE‐SPAP reporter gene and (a) the human A_2A_‐receptor or (b) the human A_2B_‐receptor. Responses to NECA are shown as red circles and VCP746 as blue circles. Data points represent mean ± SEM of five separate experiments (*n* = 5), each performed in quadruplicate. Data have been expressed as a percentage of the response to 1‐μM NECA measured in the same experiment. Curves through the data points were fitted by non‐linear regression analysis as described under Section 2. ^*^ *P* < .05 with respect to basal levels (one‐way ANOVA)

The lack of effect of the A_1_‐antagonist DPCPX on the cardiovascular responses to VCP746, and their similarity with those obtained with adenosine, suggests that VCP746 is able to stimulate other adenosine receptors in the cardiovascular system. It has previously been shown that VCP746 lacks agonist efficacy at adenosine A_3_‐receptors (Valant et al., 2014). Vecchio et al. (2016) have recently shown that VCP746 is a high affinity and potent agonist of A_2B_‐receptors. However, the potent vasodilator and tachycardia effects of VCP746 observed in the present study were more consistent with an effect on A_2A_‐receptors (Alberti et al., 1997; Borea et al., 2018; Headrick, Ashton, Meyer, & Peart, 2013). As a consequence, we have evaluated the agonist effect of VCP746 on CRE‐mediated reporter gene responses in CHO‐K1 cells stably expressing either the human A_2A_‐receptor or the human A_2B_‐receptor (Figure 9). Consistent with previous reports (Vecchio et al., 2016), VCP746 was a potent and efficacious agonist of A_2B_‐receptor‐mediated CRE‐gene expression (pEC_50_ = 8.7 ± 0.1, *E*_max_ = 103.0 ± 4.6% of response to 1‐μM NECA, *n* = 5; Figure 9; Table 2). VCP746 was, however, also a potent and high efficacy agonist of A_2A_‐receptor‐mediated CREP‐SPAP responses (pEC_50_ = 7.4 ± 0.1, *E*_max_ = 104.0 ± 15.0% of response to 1‐μM NECA, *n* = 5; Figure 9; Table 2).

**Table 2:** Concentration–response parameters for the agonist effects of NECA and VCP746 on CRE‐mediated gene expression in CHO‐K1 cells expressing either the human A_2A_‐ or A_2B_‐receptors

	A_2A_‐receptor			A_2B_‐receptor		
	pEC_50_	*E*_max_	*n*	pEC_50_	E_max_	*n*
NECA	7.50 ± 0.17	102.4 ± 6.1	5	7.29 ± 0.17	107.1 ± 2.6	5
VCP746	7.38 ± 0.07	104.0 ± 15.0	5	8.69 ± 0.11	103.0 ± 4.6	5

*Note.* Data are expressed as mean ± SEM in *n* separate experiments, performed in quadruplicate. *E*_max_ values are expressed as a percentage of the response to 1‐μM NECA measured in the same experiment.

## 4 DISCUSSION

In the present study, we have investigated the effect of a novel bitopic ligand VCP746, which has been reported to be a biased A_1_‐agonist (Valant et al., 2014), on cardiovascular responses in conscious, freely moving rats. The aim was to establish whether a hybrid molecule containing both adenosine and the positive allosteric enhancer, VCP171, could provide a mechanism to activate a subset of A_1_‐receptor‐mediated responses in particular vascular beds. The selective A_1_‐receptor agonist, CCPA, exhibited the full range of A_1_‐mediated cardiovascular responses leading to bradycardia, vasoconstriction in the renal and mesenteric vascular beds, an increase in vascular conductance in the hindquarters and a fall in mean arterial pressure. The small increase in vascular conductance in the hindquarters was only mediated at the highest dose of CCPA and could be due to activation of A_2A_‐ or A_2B_‐receptors. The decrease in this effect by DPCPX did not reach statistical significance but could be related to the known ability of DPCPX to antagonize A_2B_‐receptors (pK_B_ of 7.87; Vecchio et al., 2016). The fall in mean arterial pressure caused by CCPA was delayed compared to the effects of this A_1_‐agonist on heart rate and the vasoconstriction in the renal and mesenteric vascular beds and may well be a consequence of the similarly delayed vasodilatation in the hindquarters.

These responses, however, were very similar to those reported by Jolly et al. (2008) with another A_1_‐selective agonist (2‐methyl‐CCPA). Interestingly, the lower efficacy A_1_‐agonist, capadenoson (Albrecht‐Kupper et al., 2012; Tendera et al., 2012), produced a dose‐dependent decrease in HR (which was sensitive to inhibition by DPCPX), but this was not accompanied by significant changes in the other A_1_‐receptor‐mediated cardiovascular responses. These data suggest that adenosine A_1_‐responses in different parts of the cardiovascular system may well be subject to different signalling efficiencies.

To determine whether differences in signalling efficiency or preferential activation of particular signalling pathways (i.e., via biased‐agonism) can be exploited by a bitopic ligand, such as VCP746, we have investigated the effect of this ligand in conscious rats subject to normal autonomic reflex control. Previous work in isolated rat atria showed that VCP746 did not produce the normal A_1_‐receptor‐mediated negative chronotropic effect, but it was able to reduce ischaemic damage, hypertrophy and remodelling in cardiomyocytes (Chuo et al., 2016; Valant et al., 2014). Application of VCP746, however, produced a significant increase in HR in conscious, freely moving rats that occurred in parallel with marked increases in renal and mesenteric vascular conductance. These effects were the complete opposite of those expected of an A_1_‐receptor agonist and were not inhibited by the selective A_1_‐antagonist DPCPX.

The responses to VCP746 were in many respects similar to those elicited by adenosine, which produced tachycardia, increased vascular conductance in all three vascular beds and a substantial fall in mean arterial BP. All of these effects of adenosine were unaffected by DPCPX treatment and were very similar to those reported by Jolly et al. (2008) for adenosine using a similar conscious rat model.

VCP171, at doses that were the equimolar equivalent of those found in pharmacologically active doses of VCP746, did not produce any major changes in the responses to adenosine. VCP171 certainly did not markedly enhance the contribution of A_1_‐receptors in the final response, with the possible exception of a small effect on heart rate. VCP171 does not produce a large enhancement of the affinity of adenosine at the rat A_1_‐receptor (Cooper et al., 2019) and it is likely that the positive A_1_‐receptor allosteric action of the VCP171 entity within the larger VCP746 molecule is not sufficient to overcome the apparently more potent effect of the adenosine moiety on adenosine A_2A_‐ or A_2B_‐receptors in the cardiovascular system. This finding points to the need swap adenosine for a more A_1_‐selective agonist within any future bitopic ligands and to match carefully the concentration range over which the orthosteric and allosteric components operate. It is also worth pointing out that the probe dependence of allosteric modulators also needs to be considered (Cooper et al., 2019). For example, VCP171 does not produce a significant positive allosteric effect on the binding of capadenoson to the rat A_1_‐receptor (Cooper et al., 2019).

Cardiovascular responses to adenosine in the heart (via both A_1_‐ and A_2A_‐receptors) are complex with an A_1_‐mediated reduction in heart rate balanced by a A_2A_‐mediated increase in heart rate (Evoniuk, Jacobson, Shamim, Daly, & Wurtman, 1987; Jolly et al., 2008). It is worth noting that A_2A_‐receptors are also found in the cardiovascular regulatory regions of the brain (Thomas, St Lambert, Dashwood, & Spyer, 2000). Thus, an action at adenosine receptors in both the brain and the periphery is likely to contribute to the overall effect of systemically administered adenosine (Schindler et al., 2005). Furthermore, A_2A_‐receptor effects on the heart have been proposed to be partly not only due to a baroreceptor reflex response to the A_2A_‐receptor‐mediated vasodilatation (Alberti et al., 1997; Barraco, Janusz, Polasek, Parizon, & Roberts, 1988; Ohnishi, Biaggioni, Deray, Branch, & Jackson, 1986; Thomas et al., 2000) but also due to a direct activation of A_2A_‐receptors in the heart (Lappe, Sheldon, & Cox, 1992) and the CNS (Schindler et al., 2005). Lappe et al. (1992) have also shown that systemic administration of an A_2A_ agonist produces larger increases in heart rate in conscious animals than anaesthetized animals. It is therefore likely that in conscious animals, the A_1_‐mediated effects on heart rate are masked by a greater contribution from A_2A_‐receptors via central A_2A_‐receptors and via baroreceptive mechanisms. This balance of A_1_‐ and A_2A_‐responses (and the role of reflex mechanisms) in conscious animals may also explain the lack of effect of VCP746 on heart rate in isolated rat atria (Valant et al., 2014).

It has been shown previously that VCP746 is a potent and high efficacy agonist at adenosine A_2B_‐receptors (Vecchio et al., 2016). However, the potent vasodilator and tachycardia effects of both VCP746 and adenosine observed in the present study seem more consistent with an effect on A_2A_‐receptors (Alberti et al., 1997; Borea et al., 2018; Headrick et al., 2013). As mentioned above, A_2A_‐receptor effects on heart rate have been proposed to be due to both baroreceptor reflex responses to A_2A_‐receptor‐mediated vasodilatation (Barraco et al., 1988; Ohnishi et al., 1986; Thomas et al., 2000) and direct activation of A_2A_‐receptors in the heart (Lappe et al., 1992) and brain (Schindler et al., 2005). However, in the case of activation by VCP746, there was no change in mean arterial BP, suggesting that its effects are due to direct activation of A_2A_‐receptors in the heart and CNS or alternatively a consequence of A_2B_‐receptor activation.

To establish whether VCP746 has agonist activity at A_2A_‐receptors, in addition to its reported A_2B_‐effect, we investigated its ability to stimulate cAMP response element reporter gene responses in CHO‐K1 cells expressing either the human A_2A_‐receptor or the human A_2B_‐receptor. In keeping with previous published work (Vecchio et al., 2016), VCP746 was nearly two orders of magnitude more potent than NECA at activating A_2B_‐receptor. However, as predicted from the in vivo cardiovascular data obtained in the present manuscript, VCP746 was also a potent and high efficacy agonist of A_2A_‐mediated CRE‐SPAP responses. An agonist effect of VCP746 on cAMP formation at A_2A_‐receptors has also been reported recently in CHO cells (Aurelio et al., 2018).

In summary, the present study has shown that the bitopic ligand, VCP746, does not stimulate cardiovascular responses that are mediated by the adenosine A_1_‐receptor. In contrast, it produces a marked increase in heart rate and vascular conductance in both the renal and mesenteric circulation that were opposite to those expected of an A_1_‐agonist. Studies of cAMP‐mediated gene transcription responses in CHO‐K1 cells have confirmed that VCP746 is a potent and efficacious agonist of the adenosine A_2A_‐receptor, in addition to its known actions at the A_1_‐ and A_2B_‐receptors. Taken together, these data suggest that the cardiovascular responses to VCP746 are mediated by stimulation of cardiovascular A_2A_‐ or A_2B_‐receptors rather than A_1_‐receptors. This has implications for the design of future bitopic ligands that incorporate A_1_ allosteric ligand moieties to overcome on‐target cardiovascular side effects of drugs designed as A_1_‐agonists for the treatment of ischaemic heart disease.

## AUTHOR CONTRIBUTIONS

S.L.C., S.J.H., and J.W. participated in research design. M.J. and P.J.S. synthesized VCP171 and VCP746. S.L.C., J.M., and J.W. conducted regional haemodynamic experiments. A.R.S. conducted CRE‐SPAP in vitro experiments. S.L.C., A.R.S., S.J.H., and J.W. performed the data analysis. S.L.C., J.M., S.J.H., P.J.S., and J.W. wrote or contributed to the writing of the manuscript.

## CONFLICT OF INTEREST

The authors declare no conflict of interest.

## DECLARATION OF TRANSPARENCY AND SCIENTIFIC RIGOUR

This Declaration acknowledges that this paper adheres to the principles for transparent reporting and scientific rigour of preclinical research as stated in the *BJP* guidelines for https://bpspubs.onlinelibrary.wiley.com/doi/full/10.1111/bph.14207 and https://bpspubs.onlinelibrary.wiley.com/doi/full/10.1111/bph.14206, and as recommended by funding agencies, publishers and other organisations engaged with supporting research.
